# An ex-vivo and in-vitro dynamic simulator for surgical and transcatheter mitral valve interventions

**DOI:** 10.1007/s11548-023-03036-4

**Published:** 2023-12-08

**Authors:** Roger Karl, Gabriele Romano, Josephin Marx, Matthias Eden, Philipp Schlegel, Lubov Stroh, Samantha Fischer, Maximilian Hehl, Reinald Kühle, Lukas Mohl, Matthias Karck, Norbert Frey, Raffaele De Simone, Sandy Engelhardt

**Affiliations:** 1 Ruprecht-Karls University of Heidelberg, Heidelberg, Germany; 2 Department of Cardiac Surgery, Heidelberg University Hospital, Heidelberg, Germany; 3 Department of Internal Medicine III, Heidelberg University Hospital, Im Neuenheimer Feld 410, 69120 Heidelberg, Germany; 4 German Center for Cardiovascular Research (DZHK), Partner Site Heidelberg/Mannheim, Heidelberg, Germany; 5 Department of Anesthesiology, Heidelberg University Hospital, Heidelberg, Germany; 6 Clinic and Polyclinic for Oral and Maxillofacial Surgery, Heidelberg University Hospital, Heidelberg, Germany

**Keywords:** Minimally invasive mitral valve surgery, Transcatheter edge-to-edge repair, Dynamic physical simulator, Patient-specific planning, Mitral valve repair

## Abstract

**Purpose:**

Minimally invasive mitral valve surgery (MIMVS) and transcatheter edge-to-edge repair (TEER) are complex procedures used to treat mitral valve (MV) pathologies, but with limited training opportunities available. To enable training, a realistic hemodynamic environment is needed. In this work we aimed to develop and validate a simulator that enables investigation of MV pathologies and their repair by MIMVS and TEER in a hemodynamic setting.

**Methods:**

Different MVs were installed in the simulator, and pressure, flow, and transesophageal echocardiographic measurements were obtained. To confirm the simulator’s physiological range, we first installed a biological prosthetic, a mechanical prosthetic, and a competent excised porcine MV. Subsequently, we inserted two porcine MVs—one with induced chordae tendineae rupture and the other with a dilated annulus, along with a patient-specific silicone valve extracted from echocardiography with bi-leaflet prolapse. Finally, TEER and MIMVS procedures were conducted by experts to repair the MVs.

**Results:**

Systolic pressures, cardiac outputs, and regurgitations volumes (RVol) with competent MVs were 119 ± 1 mmHg, 4.78 ± 0.16 l min^−1^, and 5 ± 3 ml respectively, and thus within the physiological range. In contrast, the pathological MVs displayed increased RVols. MIMVS and TEER resulted in a decrease in RVols and mitigated the severity of mitral regurgitation.

**Conclusion:**

Ex-vivo modelling of MV pathologies and repair procedures using the described simulator realistically replicated physiological in-vivo conditions. Furthermore, we showed the feasibility of performing MIMVS and TEER at the simulator, also at patient-specific level, thus providing new clinical perspectives in terms of training modalities and personalized planning.

**Supplementary Information:**

The online version contains supplementary material available at 10.1007/s11548-023-03036-4.

## Introduction

Mitral regurgitation (MR) is the second most common valvular heart disease worldwide [1]. Chronic MR results from a broad spectrum of anatomical changes, which may selectively affect the papillary muscles, the chordae tendineae, the valve leaflets, or the annulus (primary MR) or be secondary to geometric remodeling of the ventricle (secondary MR) [2]. In symptomatic patients, surgical treatment or percutaneous interventions are required due to the failure of medical therapy or evidence of secondary organ damage. Among the available treatments, minimally invasive mitral valve surgery (MIMVS) and transcatheter edge-to-edge repair (TEER) are two of the most commonly used procedures to treat primary and secondary MR. The shift towards minimally invasive approaches leads to more complex procedures needing longer learning curves, and is dependent on high-volume centers to achieve good outcomes [3, 4]. Therefore, surgical or interventional training is gaining ground but suffers from several limitations: Access to training on live animals is costly, of ethical concern, and therefore rarely used [5]. Patient-specific planning and training to treat complex pathologies on healthy animals is impossible. Further training is currently carried out on in-vitro silicone models, reproducing only parts of the mitral apparatus and can offer only limited feedback to the user [6].

Much of the training is performed directly in the operating room by experts in high-volume centers. “See one, do one, teach one” [7, p. 1149] is often the way of learning and gaining experience in this field. Studies suggest that “see one, simulate many, do one competently, and teach everyone” [7, p. 1153] could improve the surgeon’s skill level and the patient’s outcome [7].

## Related and previous work

In our previous work, a novel method of producing patient-specific silicone mitral valves (MV) was presented. The work flow is comprised of creating a surface mesh out of segmented ultrasound data, merging the mesh with computer-designed geometries to generate a casting mold, 3D-printing the casting mold, and finally casting the MV out of silicone [8, 9]. Furthermore, the benefits for surgeons from personalized planning before the actual MIMVS were shown [10]. A dynamic environment revealing the intervention’s true benefit in restoring the valve’s functional competency is a key aspect that has, however, not been addressed to date. Gollmann-Tepeköylü et al. presented a dynamic simulator for TEER by using an entire porcine heart. Although this method allows for familiarization with TEER systems, the authors did not show realistic pressures, flows, or specific pathologies [11]. Ginty et al. developed the first dynamic patient-specific simulators for MIMVS [12] and TEER [13] interventions. While their work shows promising results, a comparison of MV behavior in physiological, pathological, and repaired conditions in terms of pressure and flow as well as validation with ex-vivo (e.g., porcine) or prosthetic MVs were not included in their study. They did not perform TEER but instead simulated its outcome with its surgical pendant, the Alfieri edge-to-edge technique [13].

In this work, we aim to develop and validate a physical simulator that, for the first time, combines the following four major aspects:

- *Anatomy* The MV apparatus, potentially patient-specific, should be integrated, including the annulus, leaflets, chordae tendineae, and papillary muscles.
- *Hemodynamic environment* The simulator should generate a hemodynamic environment for realistic behaviors in physiological, pathological, and repaired conditions for prosthetic, porcine, and patient-specific MVs.
- *Therapy* Physicians should be able to perform MIMVS in the rested simulator and TEER in the beating simulator. Therefore, the simulator should provide access to the MVs, and allow for observations and manipulations similar to those during the intervention.
- *Observations and measurements* It should be possible to perform detailed quantitative and qualitative evaluation of the hemodynamic situation via flow-, and pressure measurement, visual observation, and transesophageal echocardiography (TEE) to assess the MV and the success of repair.

Overall, this pilot study serves as a pre-clinical proof of concept evaluation, with the potential to integrate this technology into physicians’ daily routines.

## Simulator design

**Fig. 1:**
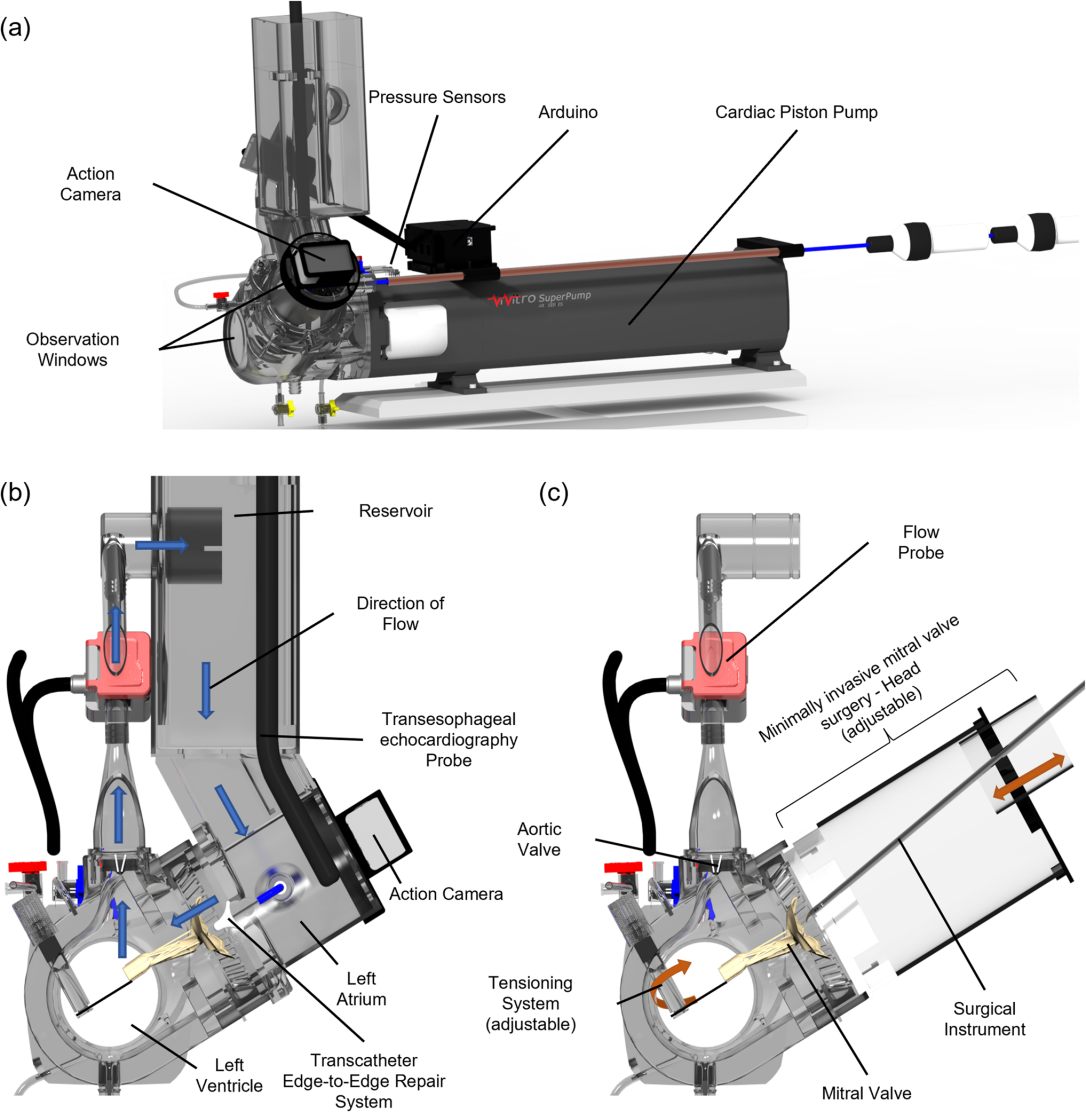
**a** Total view and **b** side cross-section view in dynamic setup for transcatheter edge-to edge repair. **c** Side cross-section view in static setup for minimally invasive mitral valve surgery

The simulator depicted in Fig. 1 is designed to replicate the function of the left heart and comprises the left atrium (LA), the left ventricle (LV), the MV, the aortic valve, and the reservoir. The latter separates the aortic valve from the LA. The *SuperPump*, a pulsatile cardiac piston pump in combination with a viscoelastic impedance adapter (both ViVitro Labs, Inc., BC, Canada), is attached to the LV and produces preselected and customizable waveforms at various frequencies and amplitudes. A compliance chamber allows for continuous pressure adjustment. Furthermore, the LA and LV have windows enabling direct observation of the simulated MV activity. The aortic valve is a 27 mm mechanical prosthetic valve (27 AHPJ-505, Abbott Laboratories, Illinois, USA). A tensioning system at the ventricle’s apex holds the papillary muscles of the MV inside the LV. This system allows continuous adjustment of position and tension force on the papillary muscles and, consequently, the chordae tendineae. The LA can incorporate a TEE probe inserted from above and a TEER-system via transseptal access (Fig. 1b). The LA can be exchanged by a tailored MIMVS-head for performing MIMVS easily and quickly resembling a right mini-thoracotomy (Fig. 1c). The MIMVS-head was designed according to the validated simulator used by Engelhardt et al. [9] and Fischer et al. [10]. A 3D endoscopic camera can be attached to perform MIMVS in a video-assisted or total endoscopic manner. The MIMVS-head, with an opening diameter of 50 mm, creates an adjustable distance of 150–155 mm between the surgeon and the annulus plane of the MV. Several parts, such as the LA, LV, MIMVS-head, and tensioning system, were custom-designed and printed from clear V4 resin on a Form 3B stereolithography printer (both Formlabs, Massachusetts, USA).

To measure cardiac output (CO), two Sonoflow CO.55 V2.0 sensors (SONOTEC GmbH, Halle (Saale), Germany) were placed between the aortic valve and the reservoir. Two HONEYWELL ABPDRRT005PG2A5 sensors (Honeywell International Inc., North Carolina, USA) measured the pressure of the LA and LV. A fluid mixture of 70% water and 30% glycerol was used to mimic blood viscosity. The ultrasound backscatter properties were simulated by adding 1% corn-starch for color-doppler measurements [14]. For echocardiographic examination during MIMVS experiments, an EPIQ CVx ultrasound system and an X8-2T 3D-TEE ultrasound probe (both Koninklijke Philips N.V., Amsterdam, Netherlands**)** were utilized. For TEER procedures, a Pascal System (Edwards Lifesciences Corporation, California, USA) and a Vivid E9 ultrasound system with a 6VT-D 3D-TEE ultrasound probe (both General Electric Company, Massachusetts, USA) were used.

## Simulator validation

### Experiments

To validate the simulator, five different MVs were tested. These included two prosthetic MVs (biological and mechanical), two explanted healthy porcine valves (ex-vivo), and a patient-specific silicone valve replica (in-vitro).

- The Biological valve was a 31 mm MV prosthesis (T510C31, Medtronic plc, Dublin, Ireland).
- The Mechanical valve was a 21 mm MV prosthesis (21 MHPJ-505, Abbott Laboratories, Illinois, USA).
- The ex-vivo valve A (for MIMVS) was sewn into a well-sized elliptical (40 mm long and 30 mm wide) frame respecting the physiological proportions of the mitral fibrous annulus.
- The ex-vivo valve B (for TEER) was sewn into an oversized circular frame to mimic annulus dilatation. (50 mm diameter).
- The in-vitro valve was generated according to Engelhardt et al. [9] from the TEE of a patient with bi-leaflet prolapse. First, the MV was segmented and a surface mesh created. After inverting the mesh, it was combined with virtual geometries to create a negative casting mold, which was subsequently 3D-printed. Additionally to the method introduced by Engelhardt et al., a gauze mesh for the leaflets and threads for the chordae tendineae were inserted before casting to reinforce the silicone. The reinforcement with fabric, which was first introduced by Ginty et al. [12], was necessary due to a small Young’s modulus (~ 0.5 MPa) and high extensibility (~ 900%) of the silicone alone. MV structures such as primary chordae tendineae have a higher Young’s modulus (~ 85 MPa) and a lower extensibility (4.3%), so an MV solely out of silicone would lead to an excessive strain during systole [15].

**Fig. 2:**
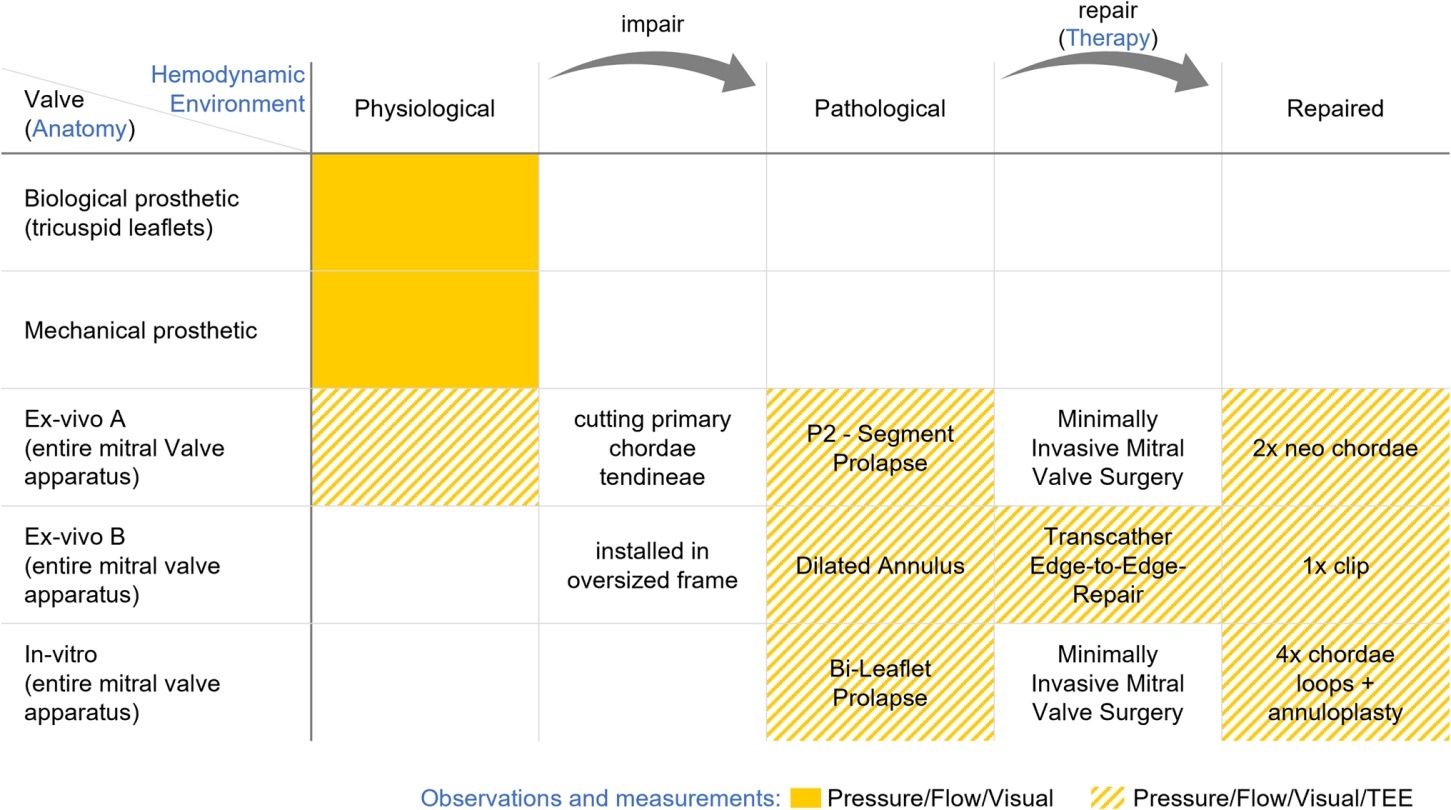
Overview of experiments. Blue text = aspects to validate. TEE, transesophageal echocardiography

Figure 2 shows the overview of the performed experiments and the conditions each MV was tested at in connection to the four aspects mentioned in the introduction. All MVs were compared at a frequency of 80 bpm and a stroke volume of 70 ml. The compliance chamber was adjusted for the physiological valves to reach a systolic blood pressure (SBP) of approximately 120 mmHg. Live monitoring was facilitated by pressure and flow sensors, and TEE 3D, B-mode, and color-doppler images were recorded.

- At first, the biological and mechanical valves were installed in the simulator to validate the simulator’s baseline at the physiological range.
- Ex-vivo valve A was then installed in a physiological state, and measurements were acquired. Primary chordae tendineae were cut at the posterior leaflet (P2–P3-segment) to induce a pathological condition. Finally, an MIMVS, consisting of two neo-chordae implantations, was performed to restore physiological MV function. During MIMVS, the LA (Fig. 1b) was replaced by the MIMVS-head (Fig. 1c).
- In the subsequent experiment, TEER was performed on the dilated MV (ex-vivo valve B) under TEE and video vision. Livestream via an action camera replaced fluoroscopy to avoid radiation. One Pascal Clip was placed between the segments A2–P2.
- Finally, the silicone patient-specific valve replica (in-vitro valve) with a bi-leaflet prolapse was tested in a pathological state and again after performing MIMVS. Specifically, a Chordae-Loop 20 mm (CV-4 needle) was implanted at the anterolateral papillary muscle, and all four loops were fixed at segments A1, A2, A3, and P1 with Cardionyl^®^ 4/0 (Peters Surgical, Boulogne-Billancourt, France). Then, after sizing, annuloplasty was performed using a 36 mm Mitral Annuloplasty Memo 4D Ring (LivaNova PLC, London, UK).

### Data acquisition and processing

The pressure and flow sensors were interfaced with the ATmega328 Microcontroller Board (AZ-Delivery Vertriebs GmbH, Germany) using the inter-integrated circuit (I2C) interface and analog current measurement, respectively. A microcontroller script was programmed to convert and transmit the data to a personal computer via USB at a frame rate of 150 frames per second. A custom Python script was used to facilitate real-time monitoring and recording of the data. The recorded data was post-processed using Matlab R2022b (The MathWorks Inc., Massachusetts, USA), and the mean values over a 30 s period were reported.

### Evaluation

The regurgitant volume (RVol) and regurgitant fraction (RF) are quantitative measurements used to grade MR. RVol refers to the backflow of fluid through the MV during the systolic phase, while RF is the ratio of the RVol to the forward flow through the MV. The RVol depends on the stroke volume set at the pump (SV_Pump_), the compression of the compliance volume (CV) after the ideal gas law, with the left ventricular end diastolic (P_LVED_) and mean systolic (P_LVMS_) absolute pressures, and the measured stroke volume after the aortic valve (SV_aortic_) and is calculated as follows:

$$ RVol = SV_{Pump} - CV\left( {1 - \frac{{P_{LVED} }}{{P_{LVMS} }}} \right) - SV_{aortic} $$

The RF is calculated from the RVol, SV_Pump_, and the CV compression as follows:

$$ RF = \frac{RVol}{{SV_{Pump} - CV\left( {1 - \frac{{P_{LVED} }}{{P_{LVMS} }}} \right)}} $$

Dujardin et al. [16] classified the severity of MR based on RVol and RF. MR with RVol < 30 ml and RF < 30% were defined as grade 1, while MR with 30–44 ml RVol and 30–39% RF were defined as grade 2, and MR with 45–59 ml RVol and 40–49% RF as grade 3. MR with even larger RVol and RF were defined as grade 4. In this study, we expanded this grading system by defining grade 0 as MR with RVol < 12 ml and RF < 19%. In spite of an expected overlap between grades, this provides a useful classification scheme [16].

MVs were categorized as physiological if they induced an SBP of 90–120 mmHg, a CO of 4.5–5 l min^−1^ and if their MR was classified as grade 0. MVs with an MR grade above 0 were defined as pathological. Successful repair was defined as MIMVS or TEER lowering the grade of MR.

## Results

Procedures were performed by a cardiac surgeon (six years in heart surgery and around 85 assisted MIMVSs), a cardio-anesthetist (two years in cardio-anesthesia and around 25 MIMVS), and two cardiologists (ten and 16 years in cardiology and both around 300 TEERs). A total of five MV models were included (Online Resource 1), on which two MIMVSs and one TEER were performed. The MVs were two prosthetic valves (biological and mechanical), two excised porcine valves (ex-vivo A, B), and one patient-specific silicone valve (in-vitro). Measurements are provided in Table 1. Three valves (biological, mechanical, and ex-vivo A) were installed in a competent condition and acted physiologically. Ex-vivo valves A and B were manipulated. Together with the in-vitro valve, in total, three valves resembled a pathological condition. The physicians successfully repaired these three valves to act competently again, which reduced the RVol and the RF by an average of 12 ± 3 ml and 17 ± 4% (Table 2).

**Table 1:** Measured parameters for all valves and experiments

	Prosthetic valves		Ex-vivo valve A			Ex-vivo valve B		In-vitro valve	
	Biological	Mechanical	Physio	Patho	Repaired	Patho	Repaired	Patho	Repaired
Compliance volume (ml)	130	130	100	100	100	40	40	0	0
Systolic blood pressure (mmHG)	118	119	120	85	117	99	106	79	115
Trans mitral gradient (mmHg)	3.21	6.69	0.72	0.72	1.49	2.32	2.51	6.86	13.5
Stroke volume (ml)	61	61	57	44	58	45	54	37	48
Cardiac output (l min^−1^)	4.88	4.90	4.55	3.49	4.67	3.56	4.3	2.98	3.83
Regurgitation volume (ml)	3	4	9	24	8	24	15	32	22
Regurgitation fraction (%)	5	6	14	35	12	35	22	47	32
Mitral regurgitation grade	0	0	0	2	0	2	1	2.3	1
P_LVMS_ (mmHg)	819	814	821	809	824	813	812	803	814
P_LVED_ (mmHG)	782	783	788	789	795	788	789	781	782

Physio., physiological; Patho., pathological; P_LVMS_, absolute mean systolic ventricular pressure; P_LVED_, absolute end diastolic ventricular pressure

**Table 2:** Flow and pressure parameters per condition

	Mitral Valves		
	Physiological	Pathological	Repaired
Systolic blood pressure (mmHg)	119 ± 1	88 ± 8	113 ± 5
Cardiac output (l min^−1^)	4.78 ± 0.16	3.34 ± 0.26	4.27 ± 0.34
Stroke volume (ml)	60 ± 2	42 ± 3	53 ± 4
Regurgitation volume (ml)	5 ± 3	27 ± 4	15 ± 5
Regurgitation fraction (%)	8 ± 4	39 ± 5	21 ± 7

All values in mean ± SD

### Biological and mechanical valve prosthesis

**Fig. 3:**
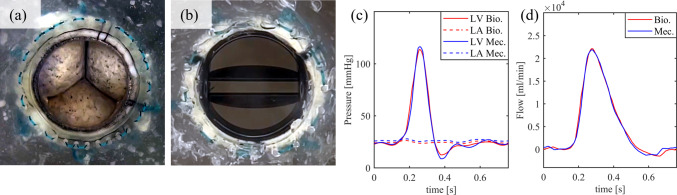
**a** Biological valve. **b** Mechanical valve and their Pressure (**c**) and Flow **d** graphs. LV, left ventricle; Bio., biological valve; LA, left atrium; Mec., mechanical valve

To determine the baseline, a realistic hemodynamic environment for MVs in physiological conditions, and the measurement of flow and pressure, the most straightforward way was to install prosthetic valves. For that purpose, the biological (Fig. 3a) and mechanical (Fig. 3b) valve prostheses were inserted in the simulator. The pressure and flow of both valves were measured. As expected, both valves demonstrated dynamic physiological behavior (Fig. 3c, d) with SBPs of 118 and 119 mmHg, COs of 4.88 and 4.90 l min^−1^, RVol of 3 and 4 ml, and RF of 5 and 6%. Therefore, the MR grade with both prosthesis is 0.

### Ex-vivo valve A for MIMVS

**Fig. 4:**
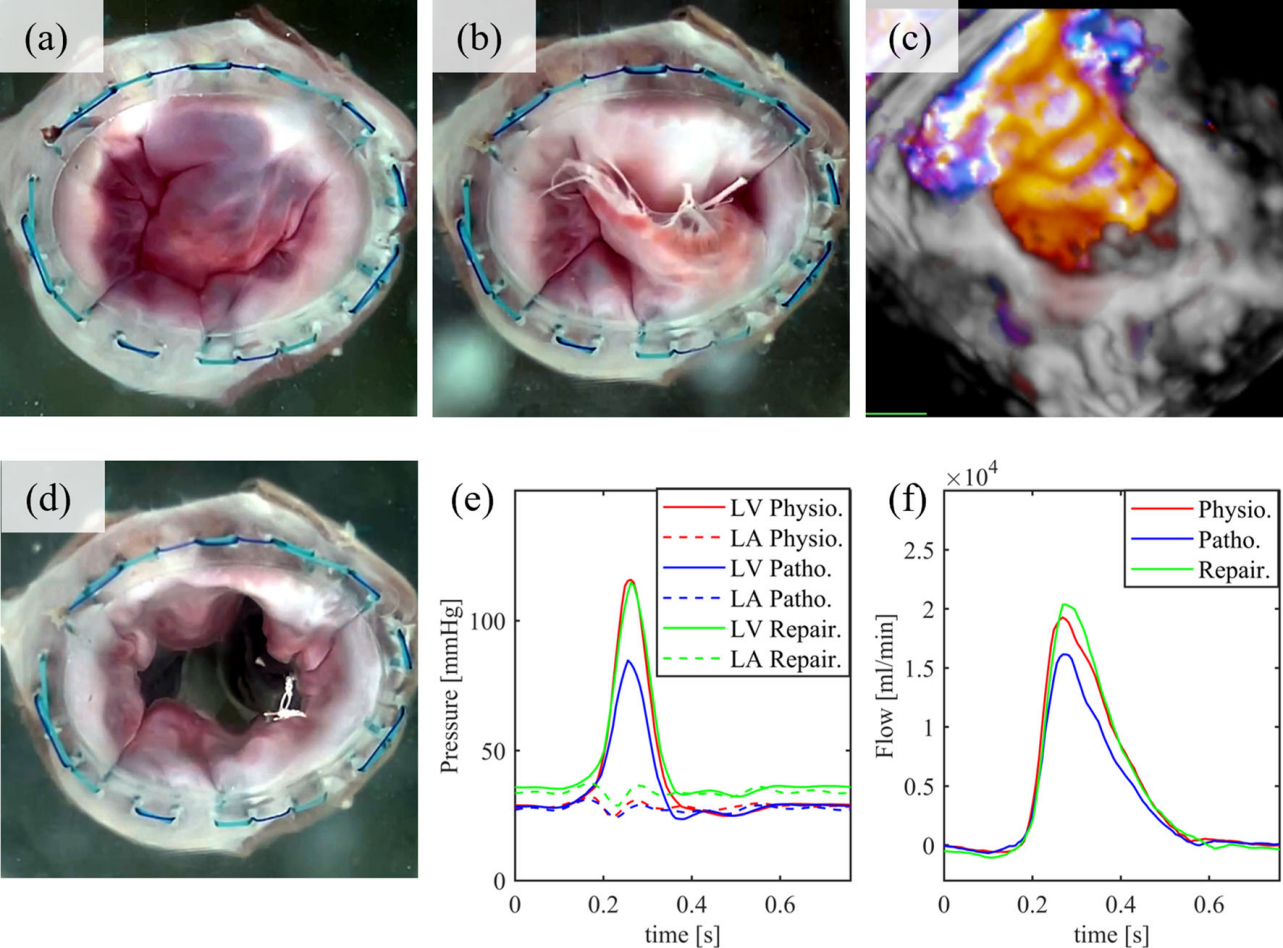
**a**–**d** Ex-vivo valve A in physiological condition (**a**), after chordae-rupture in pathological condition (**b**), 3D-color-doppler in pathological condition showing a big regurgitation jet (**c**), and after performing MIMVS in repaired condition (**d**). **e**, **f** Pressure (**e**) and Flow (**f**) graphs. LV, left ventricle; Physio., physiological condition; LA, left atrium; Patho., pathological condition; Repair., repaired condition

Furthermore, we aimed to demonstrated realistic hemodynamics with an MV in physiological, pathological, and repaired condition, as well as the integration of TEE measurements and the feasibility of performing MIMVS. The excised ex-vivo valve A consists of the whole MV apparatus, comprising the annulus, leaflets, chordae tendineae, and papillary muscles in physiological condition (Fig. 4a). Pressure, flow, and ultrasound measurements were recorded with an SBP of 120 mmHg, 4.55 l min^−1^ CO, an RVol of 9 ml, and a RF of 14%, which refers to MR grade 0. After inducing chordae rupture (Fig. 4b) by cutting primary chordae tendineae, measurements were repeated. The RVol increased to 24 ml with an RF of 35%, roughly corresponding to MR grade 2. Furthermore, a clear regurgitation jet was visible in the 3D TEE color-doppler (Fig. 4c). During MIMVS (neo-chordae insertion, Supplementary Fig. 1a) the MIMVS-head (Fig. 1c), instead of the LA (Fig. 1b), was installed. The RVol and RF with the repaired valve (Fig. 4d) decreased to 8 ml and 12%, while the SBP and the CO increased to 117 mmHg and 4.67 l min^−1^ (Fig. 4e, f). These measurements after MIMVS resembled those in the physiological state from the beginning, which showed that valve’s competency could be restored, and the MR grade was 0 again.

### Ex-vivo valve B for TEER

**Fig. 5:**
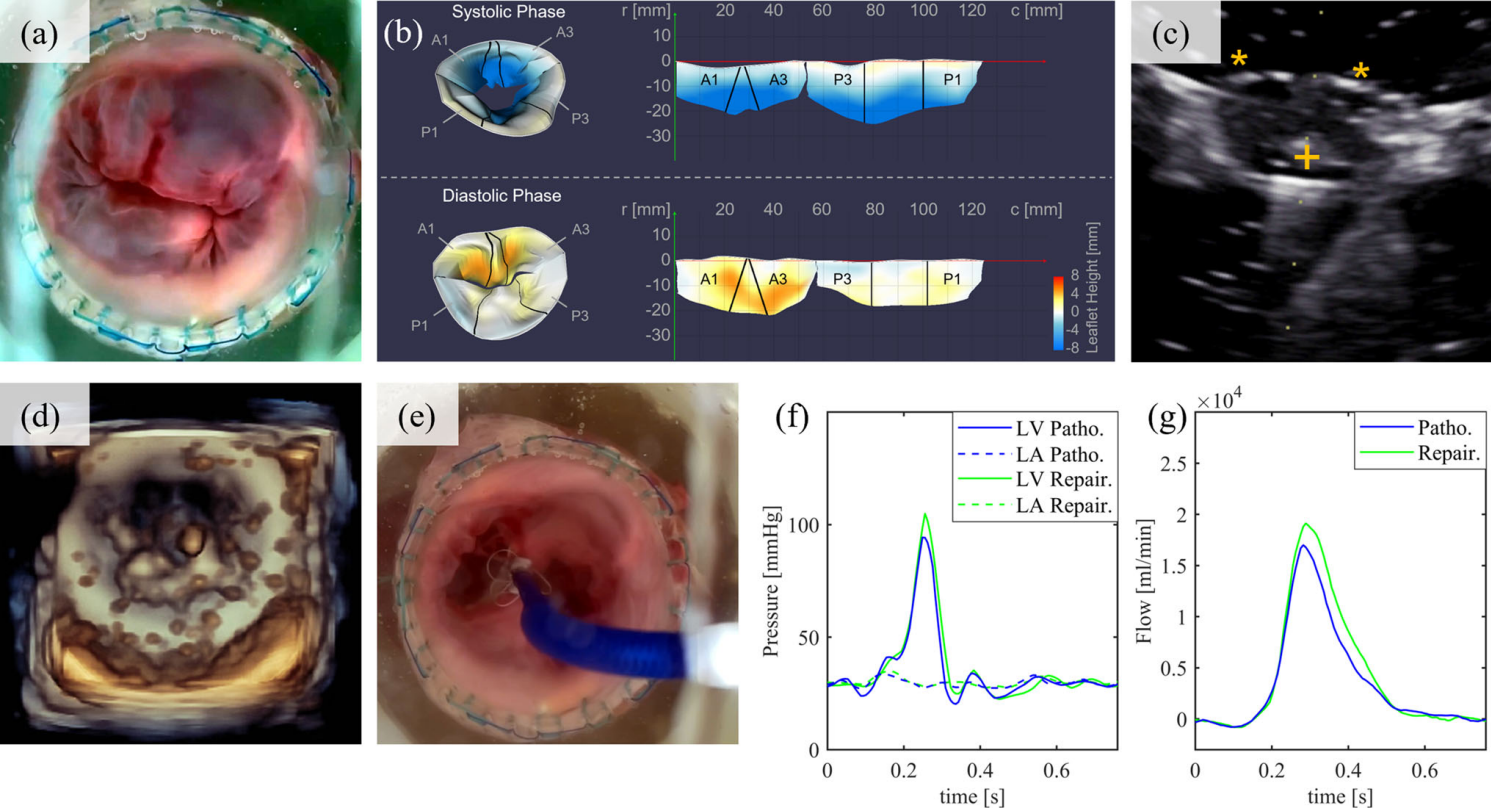
**a** Ex-vivo valve B in dilated annulus pathology. **b** 3D-view (left) and unrolled 2D-view (right) of the valve during systolic (top) and diastolic (bottom) phase. **c**, **f** B-mode (**c**) and 3D-ultrasound image (**d**) in repaired condition. **e** after TEER. **f**, **g** Pressure (**f**) and Flow (**g**) graphs. C, annulus circumference; r, radial distance to the annulus plane; A1–3, anterior leaflets; P1–3, posterior leaflets; + , clip; *, leaflet; LV, left ventricle; Patho., pathological condition LA, left atrium; Repair., repaired condition

The subsequent goal was to show the feasibility of performing TEER. To reproduce a dilated pathological condition, the ex-vivo valve B was sewed into an oversized frame (Fig. 5a). Installed in the simulator, an RVol of 24 ml and an RF of 35% were determined, predominantly corresponding to MR grade 2. The valve was segmented from TEE-data and visualized in a flattened representation (Fig. 5b), according to Lichtenberg et al. [17]. It shows an enlarged annulus (Fig. 5b top right & bottom right) of about 125 mm and displays the prolapse (Fig. 5b bottom left & bottom right (yellow/red coloring)). After the assessment of the MV, the cardiologists successfully performed TEER (Supplementary Fig. 1b). The steerable and the implant catheter were advanced into the LA via the guide sheath. The clip was oriented perpendicular to the MV coaptation, centered between the leaflets (Fig. 5c, d) in the middle of the jet seen in the 3D-TEE color-doppler. After opening the paddles and reassuring positioning, the cardiologist grasped the anterior leaflet (A2) and, afterwards, the posterior leaflet (P2) (Fig. 5e). TEER reduced the RVol to 15 ml and the RF to 22%, which means the MR grade could be reduced to 1 (Fig. 5f, g). The transmitral gradient slightly increased from 2.32 mmHg to 2.51 mmHg. Besides visual and ultrasound observations, physicians could benefit from real-time observation of pressure and flow before, during, and after the procedure. The clip was not released to enable reuse.

### In-vitro valve

**Fig. 6:**
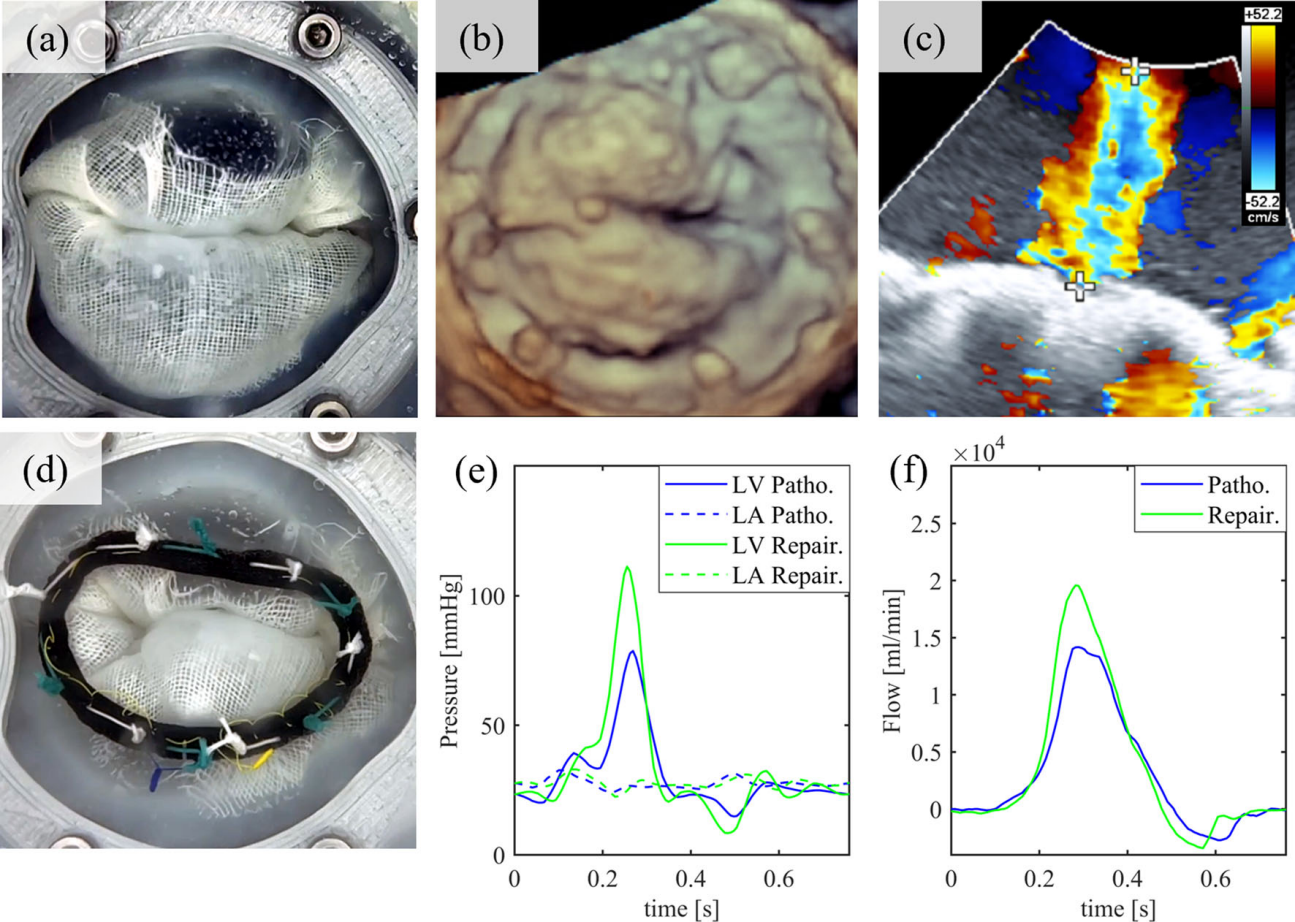
**a**–**d** In-vitro valve with bi-leaflet prolapse pathology (**a**), 3D-ultrasound image (**b**), regurgitation jet in color-Doppler mode (“+–+”) (**c**), and after MIMVS (**d**). **e**, **f** Pressure (**e**) and Flow (**f**) graphs. LV, left ventricle; Patho., pathological condition LA, left atrium; Repair., repaired condition

A patient-specific in-vitro valve with a bi-leaflet prolapse pathology (Fig. 6a, b) was installed in the simulator. A regurgitation jet could be seen in the 3D TEE color-doppler (Fig. 6c). An RVol of 32 ml and an RF of 47% were determined, corresponding to MR grades 2 and 3, respectively. After the assessment, the LA (Fig. 1b) was replaced by the MIMVS-head (Fig. 1c). The MIMVS consisted of 4 chordae loops at the segments A1, A2, A3, and P1 and an annuloplasty after sizing. Following successful MIMVS, the RVol was reduced to 22 ml and the RF to 32%, so that the repaired condition was most consistent with the criteria of MR grade 1 (Fig. 6d–f).

## Discussion

In this study, we present a left heart simulator that allows for the installation and comparison of different MV models, the assessment of their dynamics, and the simulation of interventions to treat MV pathologies. In contrast to existing simulators, it combines the following features: the generation of a realistic hemodynamic environment, the incorporation of the entire MV apparatus, the ability to perform surgical and cardiac interventions, and detailed measurement capabilities by sensors.

### Hemodynamic environment

It is crucial to replicate the in-vivo hemodynamic settings to enable a realistic training of physicians. This realistic hemodynamic environment was generated by simulators developed by Bazan et al. [18] and Paulsen et al. [19]. However, these simulators do not allow for the performance of MV repair. In contrast, simulators for MV repair introduced by Sardari et al. [20] and Gollmann-Tepeköylü et al. [11] either lack a hemodynamic environment or quantitative measurements of pressure or flow. The simulator presented here combines both, a realistic hemodynamic environment, as well as the opportunity to train MIMVS and TEER.

To create a realistic hemodynamic environment for competent MVs, we used both biological and mechanical prosthetic MVs. Additionally, the use of ex-vivo porcine valves enabled evaluation of the simulator’s functionality with human-like MVs. Both the prosthetic and porcine MVs induced physiological pressures and flows. The pathological and repaired valves accurately reproduced known in-vivo conditions with MR grades ranging from 0 to 3.

### Integration of the MV apparatus

The integration of the entire MV apparatus is essential for modelling MR pathologies and their treatment. While previously reported simulators for training and planning do not allow for the implementation of papillary muscles [12, 21], simulators solely used for research, such as the ones introduced by Paulsen et al. [19] and Rabbah et al. [22], and our simulator enable the integration of the entire MV apparatus, including the annulus, leaflets, chordae tendineae, and papillary muscles. This allowed us to model pathologies such as chordae tendineae rupture and annulus dilatation, which resulted in impaired valve competency and moderate MR (grade 2).

While simulations on whole porcine hearts, as reported by Gollmann-Tepeköylü et al. [11], allow for training of TEER only on certain pathologies or even on healthy MVs, our simulator also enables the integration of patient-specific silicone valves. Most importantly, this is a step towards facilitating pre-operative patient-specific planning in a hemodynamic environment, which is not possible with currently available simulators.

### Simulation of MIMVS and TEER

MIMVS and TEER are major therapies for treating MR, performed in cardiac surgery and cardiology, respectively. While MIMVS is performed on the arrested heart via transthoracic access, TEER is performed on the beating heart via transseptal access. Existing devices for simulating therapeutic procedures provide either transthoracic access (Sadari et al. [20]) or transseptal access (Gollmann-Tepeköylü [11]) in a realistic setup, or no realistic access at all (Boone et al. [21], Ginty et al. [12], Paulsen et al. [19]), as the MV apparatus must be completely removed from the simulator to perform procedures. In contrast, the simulator presented here realistically mimics both types of access, allowing for the simulation of both interventions in one device.

In the future, the simulator may potentially be used to practice and model additional techniques. Transseptal interventions such as the Cardioband and ChordArt could likely be integrated without design changes, whereas approaches like the Harpoon system or MitralStitch would require design modifications. However, their integration is facilitated by the modular construction of the simulator.

### Evaluation of MVs and repair performance

The feasibility to evaluate the hemodynamic condition quantitatively and qualitatively with the MV is a fundamental requirement for comparing different techniques or monitoring the training progress of a physician. Existing simulators including a hemodynamic setting either offer no flow and pressure data [11] or pressure measurements without flow data [12, 21]. In contrast, our simulator is compatible with transesophageal echocardiography (TEE) during experiments with porcine and patient-specific MVs. Moreover, additional tools are implemented to measure and monitor pressure and flow in real-time, allowing for a more detailed observation of the simulation.

Although our simulator reproduces a realistic environment for MV, and therapies to treat MR, a few aspects may be improved in the future. The tensioning system for the subvalvular apparatus should allow more adjustments due to the potential three-dimensional repositioning of papillary muscles forced by interventional influences. Furthermore, TEER training under fluoroscopy would improve the monitoring of the TEER-system once it passes the MV orifice, but would expose the physicians to radiation and would require more infrastructure for the simulation. Additionally, the rigid material of the simulator with its higher echogenicity limits the orientation and navigation in the echocardiographic image. For example, the aorta is hardly visible, but traditionally used as a reference marker by the echocardiographer.

## Conclusion

The simulated conditions before and after intervention (MIMVS and TEER) reliably reproduced known in-vivo conditions. Our approach can also be applied to patient-specific valve geometries, thus representing a major milestone toward precision medicine for surgical and interventional training and personalized planning in MV repair and paving the way for pre-procedural investigation of treatment strategies.

### Supplementary Information

Below is the link to the electronic supplementary material.

Biological and mechanical prosthetic valves in hemodynamic environment. Ex-vivo valve A in physiological and pathological condition, during minimally invasive mitral valve surgery and in repaired state. Ex-vivo valve B in pathological state, during transcatheter edge-to-edge-repair and in repaired condition. In-vitro valve in pathological and repaired state (MP4 12937 KB)

a) Cardiac surgeon performing minimally invasive mitral valve surgery at the simulator in static setup (left atrium replaced by MIMVS-head); b) Cardiologists performing transcatheter edge-to-edge repair procedure under video and ultrasound guidance (TIF 2529 KB)
