# Supplementary figures and images for: An ex-vivo and in-vitro dynamic simulator for surgical and transcatheter mitral valve interventions
Source: Int J Comput Assist Radiol Surg. 2023 Dec 8;19(3):411–21. doi: 10.1007/s11548-023-03036-4 (PMC10881771; doi:10.1007/s11548-023-03036-4)

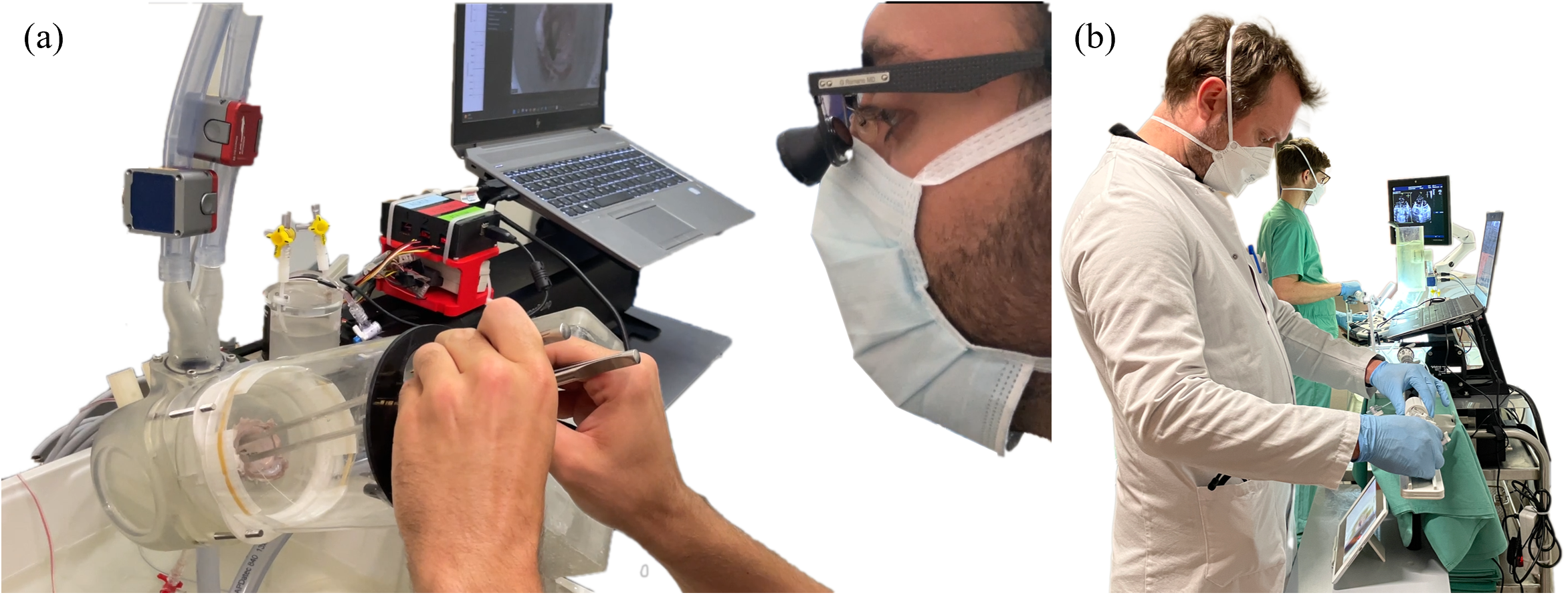

Supplement: Supplementary file 2 — a) Cardiac surgeon performing minimally invasive mitral valve surgery at the simulator in static setup (left atrium replaced by MIMVS-head); b) Cardiologists performing transcatheter edge-to-edge repair procedure under video and ultrasound guidance (TIF 2529 KB) [file 11548_2023_3036_MOESM2_ESM.tif]
